# Monetary Valuation of Congenital Heart Disease in Indonesia: Economic Modeling Study

**DOI:** 10.2196/80696

**Published:** 2025-11-19

**Authors:** Muhammad Iqhrammullah, Derren DCH Rampengan, Ikhwan Amri, Surna Lastri, Starry H Rampengan, Radityo Prakoso, Muhammad Habiburrahman

**Affiliations:** 1 Postgraduate Program of Public Health Universitas Muhammadiyah Aceh Banda Aceh Indonesia; 2 Faculty of Medicine Sam Ratulangi University Manado Indonesia; 3 Department of Geography Education Faculty of Teacher Training and Education Samudra University Langsa Indonesia; 4 Postgraduate Program of Management Universitas Muhammadiyah Aceh Banda Aceh Indonesia; 5 Division of Interventional Cardiology, Department of Cardiology and Vascular Medicine, R.D. Kandou Central General Hospital Faculty of Medicine Sam Ratulangi University Manado Indonesia; 6 Division of Paediatric Cardiology and Congenital Heart Disease, Department of Cardiology and Vascular Medicine, National Cardiovascular Centre Harapan Kita Faculty of Medicine University of Indonesia Jakarta Indonesia; 7 Faculty of Medicine Imperial College London London United Kingdom

**Keywords:** burden of disease, economic burden, health economics, health equity, pediatric cardiology

## Abstract

**Background:**

Congenital heart disease (CHD) constitutes a significant health and economic burden in low- and middle-income countries, including Indonesia. However, its macroeconomic impact across provinces remains poorly quantified.

**Objective:**

This study aims to estimate the economic burden associated with premature death and disability due to CHD in Indonesia, with a focus on regional and gender disparities.

**Methods:**

Using data from the Global Burden of Disease 2019 study, we assessed the value of lost welfare (VLW) attributable to CHD across all 34 Indonesian provinces. Economic valuation was conducted using 3 approaches: the US Department of Transportation model, a method based on the Organisation for Economic Co-operation and Development, and a national wage–based estimate. Analyses were stratified by sex and derived from both disability-adjusted life years (DALYs) and years of life lost. We examined disparities using two approaches: (1) gender disparity was measured as the relative difference in VLW between males and females, and (2) geographical disparity was quantified using both location quotients (for raw VLW) and a disparity index (for VLW-to–gross domestic product ratios).

**Results:**

The national CHD-related VLW derived from DALYs was estimated at US $16.83 billion (US Department of Transportation), US $11.41 billion (Organisation for Economic Co-operation and Development), and US $9.03 billion (wage-based). West Java recorded the highest provincial VLW (US $1.60 billion), followed by East Java (US $0.83 billion), North Sumatra (US $0.80 billion), and Central Java (US $0.79 billion), indicating a concentration of burden in populous provinces. In contrast, Yogyakarta (US $0.04 billion), North Kalimantan (US $0.04 billion), and West Papua (US $0.06 billion) had the lowest estimates. In several provinces, male-attributed VLW was more than 150% higher than female VLW, with extreme gaps observed in Riau Islands (281.25%), Aceh (166.10%), and Banten (145.86%). These patterns were consistent across both DALY- and years-of-life-lost-based estimates. Based on the location quotients, provinces such as Papua (2.42), West Sulawesi (2.37), Maluku (1.78), East Nusa Tenggara (1.75), and Central Sulawesi (1.66) bore VLW burdens far greater than their population share. The burden was disproportionately high in several eastern provinces, including East Nusa Tenggara (3.35%), Maluku (2.61%), and West Sulawesi (2.66%).

**Conclusions:**

CHD is a macroeconomically manageable burden across most of Indonesia. However, the presence of deep gender disparities and geographically concentrated burdens in eastern and underserved provinces calls for targeted pediatric cardiac health investments.

## Introduction

Despite a 56.2% global reduction in mortality, congenital heart disease (CHD) continues to cause more than 200,000 childhood deaths annually, remaining one of the leading contributors to mortality among children younger than 5 years worldwide [1]. This global progress, however, has not been equitably realized across all nations. In Indonesia, CHD-associated disability-adjusted life years (DALYs) rose from 96.3 (72.4-126.8) per 100,000 population in 2019 to 113.2 (86.6-143.7) in 2021, indicating a concerning reversal of global trends [2].

Clinical data from a national registry of 1012 pediatric patients identified secundum atrial septal defect as the predominant diagnosis, accounting for more than 70% of cases, with over three-quarters already exhibiting signs of pulmonary hypertension at the time of diagnosis [3]. The consequences of CHD extend beyond cardiovascular morbidity, encompassing significant impairments in growth and development. Previous studies have shown that children with CHD frequently present with underweight, stunting, and wasting [4]. Independent risk factors for undernutrition include cyanotic CHD, delayed diagnosis, congestive heart failure, and pulmonary hypertension [4]. In a separate cohort, nearly half of the children were aged 0-2 years, with 18.5% demonstrating developmental delays; cyanotic CHD was significantly associated with poor nutritional status [5].

Despite well-documented clinical, developmental, and survival implications, the long-term economic burden of CHD—particularly the loss of productivity due to premature mortality and disability—remains underexamined in Indonesia’s national health planning. Valuing lost life years provides a critical framework to translate disease burden into economic terms. Previous research has demonstrated the applicability of value of lost welfare (VLW) approaches to quantify the economic impact of mortality shifts in low- and middle-income countries (LMICs), reinforcing its utility in appraising health sector investments [6].

The VLW, derived from the broader concept of the value of a statistical life (VSL), provides a standardized monetary valuation for years lost to premature death or morbidity. When applied to DALYs and years of life lost (YLLs), VLW enables the conversion of epidemiological estimates into economic burden, thereby enhancing the evidence base for strategic resource allocation and policy prioritization. The increasing use of disease burden estimates in shaping national policy, such as in the European Union’s legislative decisions, underscores their practical influence [7].

Several methodologies for estimating the VSL exist, each carrying distinct policy implications. The US Department of Transportation (US DOT) model reflects valuations typical of high-income settings [8], the Organisation for Economic Co-operation and Development (OECD)–based model offers a global median, and wage-based models generate context-specific estimates more aligned with national economic realities [9]. In Indonesia, where regional disparities in income and health care access remain pronounced, disaggregating VLW by province and valuation model allows for a more nuanced understanding of both absolute and relative economic burden [10,11]. For macroeconomic assessments, Global Burden of Disease (GBD) estimates can capture regional inequalities, as reported previously in studies quantifying disparities in population health [12,13]. Previous studies have used GBD estimates to capture the trend in various causes of death in global, regional, and even national contexts [12-16]. Accordingly, this study sought to quantify the economic burden of CHD in Indonesia by estimating VLW and VLW-to–gross domestic product (GDP) per capita ratios across provinces using GBD 2019 data and 3 established VSL frameworks.

## Methods

### Ethical Considerations

This study used publicly available, deidentified data from the Global Burden of Disease 2019 study. As all data were anonymized and aggregated, no additional ethical approval was required. The data were used in accordance with the Global Burden of Disease data use policy.

### Study Design

This study quantified the economic burden associated with congenital heart anomalies (CHAs) in Indonesia by monetizing health losses using the VLW framework. We drew upon the GBD 2021 dataset, which provides comprehensive and methodologically harmonized estimates of disease burden across countries and regions. Two principal health metrics were used: DALYs—a composite of years lived with disability (YLDs) and YLLs due to premature mortality—and YLLs alone, to isolate mortality-specific economic losses. VLW was estimated by applying 3 established benchmarks of the VSL. Analyses were conducted at both national and provincial levels, stratified by sex, to examine geographic and demographic variation in the health-related economic burden.

### Data Sources

Burden estimates were extracted from GBD 2021, focusing specifically on the CHAs subcategory. YLDs were calculated by multiplying condition-specific prevalence by disability weights reflective of the severity of health loss. YLLs were estimated from mortality counts and the GBD standard life expectancy for each age group. DisMod-MR 2.1, a Bayesian meta-regression framework, was used to ensure epidemiological consistency across parameters. Additionally, Spatiotemporal Gaussian Process Regression was used to account for spatial and temporal heterogeneity in disease patterns. All estimates were disaggregated by gender and province. In provinces with limited empirical data, estimates were refined using covariate adjustments and comparisons with analogous regions. Full methodological specifications are available in the GBD technical documentation [17,18]. To contextualize the economic burden, 2021 provincial GDP per capita data were sourced from Badan Pusat Statistik (BPS, Indonesian Statistic Agency). Reported values in Indonesian Rupiah (IDR) were converted to US$ using the average 2021 exchange rate (IDR 14,308=US $1) and expressed in trillions to match the national economic scale. DALYs and YLLs of CHD and GDP per capita across Indonesian provinces are available in Multimedia Appendix 1.

### VSL Benchmarks and Economic Valuation

To monetarily quantify the societal burden associated with premature mortality from CHAs, we applied 3 benchmark estimates of the VSL. The primary benchmark was a nationally derived, wage-based VSL specific to Indonesia, estimated at US $950,000. This estimate reflects labor market trade-offs between income and occupational fatality risk, as captured through a hedonic wage model [19]. The model used data from the 2019 Survei Angkatan Kerja Nasional National (Labor Force Survey) conducted by BPS, alongside occupation-specific fatality rates provided by the Badan Penyelenggara Jaminan Sosial Kesehatan Ketenagakerjaan (Social Security Administrator for Labor) [20,21].

Multivariable linear regression was used to estimate wage differentials associated with varying levels of occupational risk, adjusting for confounding factors including industry classification, educational attainment, and demographic characteristics. To address potential selection bias—where individuals may self-select into higher-risk or better-compensated occupations—a Heckman [22] 2-stage correction model was applied. The choice of 2019 as the base year was intentional, ensuring that VSL estimates reflect stable labor market conditions before disruptions caused by the COVID-19 pandemic.

Two additional benchmarks were adapted from international reference values originating in high-income countries: one from the OECD and another from the US DOT. Both were recalibrated to the Indonesian context using the income transfer method, which adjusts VSL values according to relative national income levels. This ensures that the transferred VSLs remain consistent with Indonesia’s economic reality. Specifically, the recalibrated values were derived using the standard benefit transfer formula (equation 1):


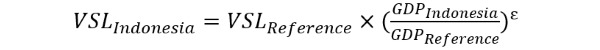
 (1)


In this equation VSL_Indonesia_ denotes the income-adjusted value of a statistical life for Indonesia; VSL_Reference_ corresponds to the original benchmark value from either the OECD or the US DOT; GDP_Indonesia_ and GDP_Reference_ represent per capita GDP values (in purchasing power parity terms) for Indonesia and the reference country, respectively; and ε (elasticity coefficient) indicates the income elasticity of VSL. Following established methodological practice for LMIC settings, we assumed an ε of 1.0, consistent with World Bank and OECD recommendations for cross-national health valuation exercises.

Using 2021 GDP per capita data, the income-adjusted VSL estimates for Indonesia were calculated as US $1.2 million (based on the OECD benchmark) and US $1.77 million (from the US DOT benchmark). These adjusted values reflect economic conditions specific to Indonesia while preserving the relative scale of the original reference values. Each of the 3 benchmark VSLs—including the nationally derived, wage-based VSL—was subsequently transformed into a VLW by dividing the respective VSL by the standard age-specific life expectancy at death, enabling alignment with GBD metrics. These VLWs were applied to DALY- and YLL-derived estimates of CHAs to produce monetized estimates of VLW, stratified by province and sex. This dual disaggregation enabled a granular evaluation of the economic burden and its association with geographic and demographic disparities.

### Monetization of DALYs and YLLs Through VLW

To quantify the economic burden associated with CHAs in Indonesia, we used a monetization approach anchored in the concept of the VLW, applying it to both DALYs and YLLs in absolute counts. To convert DALY and YLL rates into absolute counts, we multiplied them by the population estimated by the GBD (Multimedia Appendix 1). This approach leverages DALYs and YLLs as proxies for health-adjusted life years lost, facilitating economic valuation based on the VSL framework [23,24]. Following established methodological conventions [24,25], we used the inherent age stratification provided by the GBD study to retain age-specific granularity in estimating disease burden. For each province (*i*), sex (*s*), and valuation model (*m*), we derived VLW estimates by multiplying age-disaggregated DALYs or YLLs with a standardized value of a statistical life year (VSLY), which was computed by dividing VSL by life expectancy at age of death. We used separate life expectancy estimates for males and females, allowing sex-specific adjustment of VSLY in accordance with the demographic profile of each death. According to BPS, the average life expectancy in 2021 was 73.5 years for the total population, with sex-specific values of 60.67 years for males and 73.55 years for females. Three VSLY estimates were applied, corresponding to the VSL benchmarks sourced from the US DOT, the OECD, and the nationally derived wage-based estimate. The economic valuation formula is expressed as follows (equations 2 and 3):


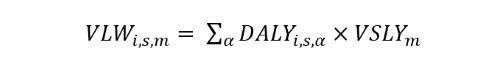
 (2)


or,


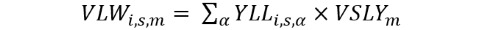
 (3)


Where α denotes the age group; DALY*_i,s,α_* or YLL*_i,s,α_* represent the disease burden (ie, DALYs or YLLs) in age group α for province *i* and sex *s* and VSLY*_m_* corresponds to the life-year valuation under model 𝑚 (USDOT, OECD, or wage-based). Finally, to contextualize these figures within local economic capacity, we calculated the VLW-to-GDP ratio for each province, defined as follows (equation 4):


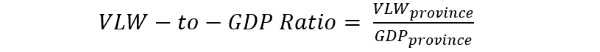
 (4)


### Disparity Estimation

We estimated gender and geographic disparities in the economic impact of CHD across Indonesia. Gender disparity was quantified using the male–female relative difference in VLW, expressed as a percentage (equation 5). This gender disparity metric reflects the proportional excess burden among males relative to females. Geographic disparity was assessed using 2 measures. First, we calculated the location quotient (LQ), defined as the ratio of a province’s share of national VLW to its share of the national population (equation 6). This metric is suitable for raw VLW data because both the numerator and denominator represent shares of national totals, allowing a valid comparison of economic burden relative to population size. An LQ greater than 1 indicates that a province bears a disproportionate share of VLW relative to its population. Second, we computed a disparity index by dividing the provincial VLW-to-GDP ratio by the national average. This approach is more appropriate for ratio-based metrics such as VLW-to-GDP, as using an LQ on these values can lead to inflated results due to the low national reference value. The disparity index quantifies how much greater (or lesser) the economic burden is in a province relative to the national benchmark, offering a stable basis for interprovincial comparisons.


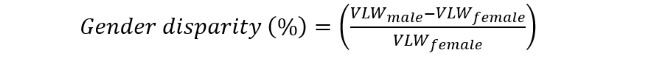
 (5)



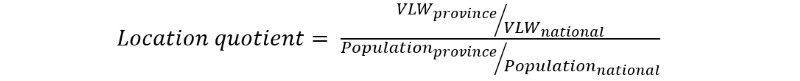
 (6)



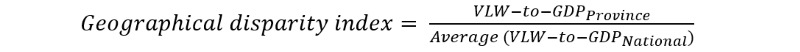
 (7)


### Data Processing and Visualization

All calculations and data integration were performed using Python 3.11, with the Pandas library used for structured data manipulation, including the aggregation and merging of health burden estimates (from GBD 2021) and provincial economic indicators. Final estimates of VLW were expressed in billion US$, computed under both DALY- and YLL-based paradigms, and stratified by sex (male, female, and combined) to reflect sex-specific differentials in health-related economic loss. To classify provinces by levels of disparity, we applied k-means clustering on both gender and geographic disparity indices, allowing unsupervised grouping into quartile-based clusters. To visually convey the geographic heterogeneity of the burden, we used Tableau Desktop 2024.1 (Tableau Software, LLC) to develop spatially resolved choropleth maps, highlighting provincial-level VLW and VLW-to-GDP ratios. This visual framework enabled the identification of regions exhibiting disproportionately high economic losses relative to their GDP, facilitating targeted policy interpretation and public health planning.

## Results

### Economic Burden Based on DALY-Derived Estimates

The DALY-based VLW estimates for CHD in Indonesia are presented in Table 1. At the national level, the total VLW burden reaches US $16.825 billion using the US DOT benchmark, US $11.407 billion using the OECD benchmark, and US $9.03 billion using the national wage benchmark. Based on the national wage–based estimates, West Java reported the highest provincial VLW at US $1.599 billion, followed by East Java (US $0.831 billion), North Sumatra (US $0.797 billion), and Central Java (US $0.793 billion). Together, these 4 provinces accounted for over 44% of the national burden. Other notable contributors include Banten (US $0.475 billion), Papua (US $0.381 billion), and South Sumatra (US $0.363 billion). In contrast, provinces such as North Kalimantan (US $0.036 billion), Yogyakarta (US $0.042 billion), and West Papua (US $0.055 billion) reported the lowest VLW.

**Table 1:** Economic burden of congenital heart disease in Indonesia estimated from disability-adjusted life year (DALY), using the US Department of Transportation (US DOT), Organisation for Economic Co-operation and Development (OECD), and wage-based models (value of lost welfare [VLW] in billion US$).

Province	VLW (US DOT)				VLW (OECD)				VLW (national wage)		
	Both	Males	Females	Both		Males	Females	Both		Males	Females
Aceh	0.352	0.293	0.110	0.239		0.199	0.075	0.189		0.157	0.059
Bali	0.179	0.121	0.079	0.121		0.082	0.053	0.096		0.065	0.042
Bangka-Belitung Islands	0.116	0.089	0.043	0.079		0.060	0.029	0.062		0.048	0.023
Banten	0.886	0.720	0.292	0.601		0.488	0.198	0.475		0.386	0.157
Bengkulu	0.127	0.101	0.044	0.086		0.068	0.030	0.068		0.054	0.024
Central Java	1.477	1.123	0.550	1.002		0.761	0.373	0.793		0.603	0.295
Central Kalimantan	0.210	0.141	0.093	0.142		0.096	0.063	0.113		0.076	0.050
Central Sulawesi	0.306	0.243	0.106	0.208		0.165	0.072	0.164		0.130	0.057
East Java	1.548	1.182	0.572	1.049		0.801	0.388	0.831		0.634	0.307
East Kalimantan	0.196	0.149	0.073	0.133		0.101	0.049	0.105		0.080	0.039
East Nusa Tenggara	0.574	0.405	0.240	0.389		0.275	0.162	0.308		0.217	0.129
Gorontalo	0.146	0.103	0.061	0.099		0.070	0.041	0.078		0.055	0.033
Indonesia	16.825	12.917	6.164	11.407		8.753	4.179	9.030		6.931	3.309
Jakarta	0.356	0.281	0.125	0.241		0.190	0.084	0.191		0.151	0.067
Jambi	0.183	0.139	0.068	0.124		0.094	0.046	0.098		0.075	0.037
Lampung	0.572	0.465	0.189	0.388		0.315	0.128	0.307		0.250	0.101
Maluku	0.209	0.145	0.090	0.142		0.098	0.061	0.112		0.078	0.048
North Kalimantan	0.067	0.053	0.024	0.046		0.036	0.016	0.036		0.028	0.013
North Maluku	0.162	0.122	0.061	0.110		0.083	0.042	0.087		0.065	0.033
North Sulawesi	0.201	0.167	0.063	0.136		0.113	0.043	0.108		0.090	0.034
North Sumatra	1.486	1.209	0.488	1.007		0.819	0.331	0.797		0.649	0.262
Papua	0.710	0.569	0.241	0.482		0.385	0.163	0.381		0.305	0.129
Riau	0.352	0.287	0.114	0.238		0.195	0.077	0.189		0.154	0.061
Riau Islands	0.122	0.113	0.029	0.083		0.076	0.020	0.065		0.061	0.016
South Kalimantan	0.324	0.245	0.122	0.220		0.166	0.083	0.174		0.132	0.066
South Sulawesi	0.608	0.475	0.216	0.412		0.322	0.147	0.326		0.255	0.116
South Sumatra	0.676	0.531	0.238	0.458		0.360	0.161	0.363		0.285	0.128
Southeast Sulawesi	0.326	0.262	0.110	0.221		0.178	0.075	0.175		0.141	0.059
West Java	2.979	2.219	1.147	2.020		1.504	0.778	1.599		1.191	0.616
West Kalimantan	0.295	0.189	0.139	0.200		0.128	0.094	0.158		0.101	0.075
West Nusa Tenggara	0.403	0.295	0.160	0.273		0.200	0.108	0.216		0.158	0.086
West Papua	0.103	0.076	0.040	0.070		0.051	0.027	0.055		0.041	0.022
West Sulawesi	0.209	0.153	0.083	0.142		0.104	0.056	0.112		0.082	0.044
West Sumatra	0.285	0.192	0.127	0.193		0.130	0.086	0.153		0.103	0.068
Yogyakarta	0.078	0.060	0.028	0.053		0.041	0.019	0.042		0.032	0.015

### Economic Burden Based on YLL-Derived Estimates

Indonesia’s overall estimated VLW reaches US $16.13 billion using the US DOT benchmark, US $10.936 billion under the OECD benchmark, and US $8.658 billion based on the national wage model (Table 2). To reflect local relevance, the national wage benchmark is prioritized in the following interpretation. The highest provincial VLW was observed in West Java (US $1.533 billion), followed by East Java (US $0.777 billion), North Sumatra (US $0.776 billion), and Central Java (US $0.742 billion). Collectively, these 4 provinces contributed more than 40% of the national burden. Other significant contributors include Banten (US $0.459 billion), South Sumatra (US $0.351 billion), and Papua (US $0.374 billion). In contrast, provinces such as North Kalimantan (US $0.035 billion), Yogyakarta (US $0.037 billion), and West Papua (US $0.054 billion) reported the lowest VLW estimates (Table 2).

**Table 2:** Economic burden of congenital heart disease in Indonesia estimated from years of life lost (YLL), using the United States Department of Transportation (US DOT), Organisation for Economic Co-operation and Development (OECD), and wage-based models (value of lost welfare [VLW] in billion US$).

Province	VLW (US DOT)				VLW (OECD)				VLW (national wage)		
	Both	Males	Females	Both		Males	Females	Both		Males	Females
Aceh	0.339	0.285	0.104	0.230		0.193	0.070	0.182		0.153	0.056
Bali	0.167	0.114	0.073	0.113		0.077	0.050	0.090		0.061	0.039
Bangka-Belitung Islands	0.112	0.086	0.041	0.076		0.058	0.028	0.060		0.046	0.022
Banten	0.856	0.701	0.278	0.580		0.475	0.188	0.459		0.376	0.149
Bengkulu	0.122	0.098	0.042	0.083		0.066	0.028	0.066		0.052	0.022
Central Java	1.383	1.063	0.506	0.938		0.720	0.343	0.742		0.570	0.272
Central Kalimantan	0.203	0.137	0.090	0.137		0.093	0.061	0.109		0.073	0.048
Central Sulawesi	0.299	0.238	0.102	0.202		0.161	0.069	0.160		0.128	0.055
East Java	1.447	1.117	0.525	0.981		0.757	0.356	0.777		0.599	0.282
East Kalimantan	0.186	0.143	0.068	0.126		0.097	0.046	0.100		0.077	0.037
East Nusa Tenggara	0.560	0.396	0.233	0.379		0.269	0.158	0.300		0.213	0.125
Gorontalo	0.143	0.101	0.060	0.097		0.068	0.040	0.077		0.054	0.032
Indonesia	16.130	12.472	5.837	10.936		8.451	3.957	8.658		6.692	3.134
Jakarta	0.330	0.264	0.112	0.224		0.179	0.076	0.177		0.142	0.060
Jambi	0.174	0.133	0.064	0.118		0.090	0.044	0.094		0.071	0.035
Lampung	0.550	0.450	0.178	0.373		0.305	0.121	0.295		0.241	0.096
Maluku	0.204	0.141	0.088	0.139		0.096	0.059	0.110		0.076	0.047
North Kalimantan	0.066	0.052	0.023	0.044		0.035	0.016	0.035		0.028	0.012
North Maluku	0.159	0.120	0.060	0.108		0.081	0.041	0.085		0.064	0.032
North Sulawesi	0.194	0.162	0.060	0.132		0.110	0.041	0.104		0.087	0.032
North Sumatra	1.446	1.183	0.469	0.980		0.802	0.318	0.776		0.635	0.252
Papua	0.696	0.559	0.235	0.472		0.379	0.159	0.374		0.300	0.126
Riau	0.335	0.277	0.107	0.227		0.188	0.072	0.180		0.149	0.057
Riau Islands	0.116	0.109	0.026	0.079		0.074	0.018	0.062		0.058	0.014
South Kalimantan	0.314	0.239	0.118	0.213		0.162	0.080	0.169		0.128	0.063
South Sulawesi	0.584	0.460	0.205	0.396		0.312	0.139	0.313		0.247	0.110
South Sumatra	0.655	0.517	0.228	0.444		0.350	0.155	0.351		0.277	0.122
Southeast Sulawesi	0.320	0.258	0.107	0.217		0.175	0.072	0.172		0.138	0.057
West Java	2.856	2.141	1.089	1.936		1.451	0.738	1.533		1.149	0.584
West Kalimantan	0.281	0.180	0.133	0.191		0.122	0.090	0.151		0.097	0.071
West Nusa Tenggara	0.390	0.286	0.153	0.264		0.194	0.104	0.209		0.154	0.082
West Papua	0.100	0.074	0.039	0.068		0.050	0.026	0.054		0.040	0.021
West Sulawesi	0.206	0.151	0.081	0.139		0.102	0.055	0.110		0.081	0.043
West Sumatra	0.271	0.182	0.120	0.183		0.124	0.081	0.145		0.098	0.064
Yogyakarta	0.068	0.054	0.023	0.046		0.037	0.016	0.037		0.029	0.012

In most provinces, the DALY-based values exceeded YLL-based values by less than 5%. For instance, the gaps were 2% in Papua, Maluku, North Maluku, Southeast Sulawesi, and West Sulawesi; 3% in Central Sulawesi, Central Kalimantan, East Nusa Tenggara, North Kalimantan, South Kalimantan, and West Nusa Tenggara; and 4% in Aceh, Bengkulu, Lampung, South Sulawesi, and West Java. Only a few provinces demonstrated higher differences, such as Bali (6%), Central Java (6%), East Java (6%), Jakarta (7%), and Yogyakarta, which had the largest gap at 12%. Nationally, the average DALY–YLL gap was approximately 4%.

### Productivity Loss Based on DALYs

The estimated CHD-related economic burden in Indonesia stands at 1.35% of GDP using the US DOT benchmark, 0.91% under the OECD benchmark, and 0.72% using the national wage benchmark (Table 3). Provincial-level insights using the national wage benchmark reveal that East Nusa Tenggara (3.08%), West Sulawesi (2.47%), Maluku (2.40%), and Gorontalo (1.93%) bear the highest CHD-related economic burden relative to their regional GDP. In contrast, Jakarta (0.07%), East Kalimantan (0.13%), Riau (0.22%), and Riau Islands (0.23%) report the lowest VLW-to-GDP ratios. Other populous provinces such as West Java (0.76%), Central Java (0.58%), and East Java (0.35%) fall at or below the national average.

**Table 3:** Value of lost welfare (VLW)–to-gross domestic product (GDP) ratios for congenital heart disease in Indonesia, based on the United States Department of Transportation (US DOT), Organisation for Economic Co-operation and Development (OECD), and wage-based valuation models (VLW-to-GDP, %).

Province		VLW (US DOT), %				VLW (OECD), %				VLW (national wage), %		
		Both	Males	Females	Both		Males	Females	Both		Males	Females
**Estimated from DALY (VLW_DALY_-to-GDP^a^)**												
	Aceh	2.01	2.75	1.26	1.36		1.87	0.85	1.08		1.48	0.68
	Bali	0.82	0.90	0.73	0.55		0.61	0.50	0.44		0.48	0.39
	Bangka-Belitung Islands	1.43	1.75	1.10	0.97		1.18	0.74	0.77		0.94	0.59
	Banten	1.40	1.85	0.94	0.95		1.25	0.64	0.75		0.99	0.50
	Bengkulu	1.67	2.11	1.21	1.13		1.43	0.82	0.90		1.13	0.65
	Central Java	1.08	1.35	0.80	0.73		0.91	0.54	0.58		0.72	0.43
	Central Kalimantan	1.25	1.33	1.17	0.85		0.90	0.79	0.67		0.71	0.63
	Central Sulawesi	1.13	1.43	0.81	0.77		0.97	0.55	0.61		0.77	0.44
	East Java	0.65	0.82	0.48	0.44		0.56	0.32	0.35		0.44	0.26
	East Kalimantan	0.25	0.30	0.19	0.17		0.20	0.13	0.13		0.16	0.10
	East Nusa Tenggara	5.74	6.65	4.83	3.89		4.51	3.27	3.08		3.57	2.59
	Gorontalo	3.59	4.05	3.12	2.43		2.74	2.11	1.93		2.17	1.67
	Indonesia	0.13	0.16	0.09	0.09		0.11	0.06	0.07		0.09	0.05
	Jakarta	0.79	0.97	0.59	0.53		0.66	0.40	0.42		0.52	0.32
	Jambi	1.61	2.10	1.09	1.09		1.42	0.74	0.86		1.12	0.58
	Lampung	4.47	4.98	3.95	3.03		3.37	2.68	2.40		2.67	2.12
	Maluku	0.56	0.68	0.41	0.38		0.46	0.28	0.30		0.37	0.22
	North Kalimantan	2.72	3.26	2.15	1.84		2.21	1.46	1.46		1.75	1.16
	North Maluku	1.48	1.96	0.98	1.00		1.33	0.66	0.79		1.05	0.52
	North Sulawesi	1.84	2.46	1.21	1.24		1.67	0.82	0.99		1.32	0.65
	North Sumatra	2.94	3.64	2.13	1.99		2.47	1.45	1.58		1.95	1.14
	Papua	0.42	0.55	0.28	0.28		0.38	0.19	0.22		0.30	0.15
	Riau	0.42	0.63	0.20	0.28		0.43	0.14	0.23		0.34	0.11
	Riau Islands	1.56	1.92	1.18	1.06		1.30	0.80	0.84		1.03	0.64
	South Kalimantan	1.16	1.50	0.82	0.79		1.02	0.56	0.62		0.81	0.44
	South Sulawesi	1.35	1.71	0.98	0.92		1.16	0.66	0.73		0.92	0.52
	South Sumatra	2.46	3.20	1.70	1.67		2.17	1.15	1.32		1.72	0.91
	Southeast Sulawesi	1.42	1.72	1.12	0.97		1.17	0.76	0.76		0.92	0.60
	West Java	1.32	1.34	1.30	0.90		0.91	0.88	0.71		0.72	0.70
	West Kalimantan	3.10	3.71	2.49	2.10		2.51	1.69	1.66		1.99	1.34
	West Nusa Tenggara	1.30	1.48	1.11	0.88		1.00	0.75	0.70		0.79	0.59
	West Papua	4.60	5.41	3.75	3.12		3.67	2.55	2.47		2.90	2.02
	West Sulawesi	1.16	1.28	1.04	0.79		0.87	0.70	0.62		0.69	0.56
	West Sumatra	2.01	2.75	1.26	1.36		1.87	0.85	1.08		1.48	0.68
	Yogyakarta	0.82	0.90	0.73	0.55		0.61	0.50	0.44		0.48	0.39
**Estimated from YLL (VLW_YLL_-to-GDP^b^)**												
	Aceh	2.35	3.93	1.44	1.59		2.67	0.98	1.26		2.11	0.77
	Bali	0.95	1.29	0.84	0.65		0.87	0.57	0.51		0.69	0.45
	Bangka-Belitung Islands	1.67	2.49	1.25	1.13		1.69	0.85	0.90		1.34	0.67
	Banten	1.64	2.63	1.09	1.11		1.78	0.74	0.88		1.41	0.58
	Bengkulu	1.93	3.00	1.36	1.31		2.04	0.92	1.04		1.61	0.73
	Central Java	1.24	1.89	0.91	0.84		1.28	0.62	0.67		1.02	0.49
	Central Kalimantan	1.44	1.87	1.33	0.98		1.26	0.90	0.77		1.00	0.72
	Central Sulawesi	1.32	2.04	0.93	0.90		1.38	0.63	0.71		1.10	0.50
	East Java	0.74	1.15	0.54	0.50		0.78	0.37	0.40		0.62	0.29
	East Kalimantan	0.29	0.42	0.22	0.20		0.29	0.15	0.16		0.23	0.12
	East Nusa Tenggara	6.79	9.61	5.64	4.60		6.51	3.82	3.64		5.16	3.03
	Gorontalo	4.25	5.90	3.60	2.88		4.00	2.44	2.28		3.17	1.93
	Indonesia	1.35	2.06	0.99	0.91		1.40	0.67	0.72		1.11	0.53
	Jakarta	0.15	0.23	0.10	0.10		0.16	0.07	0.08		0.12	0.05
	Jambi	0.91	1.36	0.68	0.61		0.92	0.46	0.49		0.73	0.37
	Lampung	1.88	3.00	1.25	1.28		2.03	0.85	1.01		1.61	0.67
	Maluku	5.28	7.22	4.59	3.58		4.89	3.11	2.84		3.87	2.46
	North Kalimantan	0.67	1.00	0.50	0.46		0.68	0.34	0.36		0.54	0.27
	North Maluku	3.20	4.71	2.48	2.17		3.19	1.68	1.72		2.52	1.33
	North Sulawesi	1.72	2.81	1.10	1.17		1.90	0.74	0.92		1.51	0.59
	North Sumatra	2.16	3.53	1.40	1.46		2.39	0.95	1.16		1.90	0.75
	Papua	3.45	5.21	2.49	2.34		3.53	1.69	1.85		2.80	1.34
	Riau	0.49	0.79	0.32	0.33		0.54	0.22	0.26		0.42	0.17
	Riau Islands	0.50	0.92	0.23	0.34		0.62	0.16	0.27		0.49	0.12
	South Kalimantan	1.82	2.72	1.38	1.23		1.84	0.93	0.98		1.46	0.74
	South Sulawesi	1.36	2.15	0.94	0.92		1.46	0.64	0.73		1.16	0.51
	South Sumatra	1.60	2.47	1.13	1.08		1.67	0.77	0.86		1.32	0.61
	Southeast Sulawesi	2.91	4.63	1.98	1.98		3.14	1.34	1.56		2.48	1.06
	West Java	1.67	2.47	1.29	1.13		1.67	0.88	0.90		1.32	0.69
	West Kalimantan	1.55	1.93	1.51	1.05		1.30	1.02	0.83		1.03	0.81
	West Nusa Tenggara	3.60	5.28	2.84	2.44		3.58	1.92	1.93		2.83	1.52
	West Papua	1.53	2.15	1.27	1.04		1.45	0.86	0.82		1.15	0.68
	West Sulawesi	5.44	7.85	4.36	3.69		5.32	2.96	2.92		4.21	2.34
	West Sumatra	1.34	1.79	1.19	0.91		1.21	0.81	0.72		0.96	0.64
	Yogyakarta	0.58	0.93	0.39	0.39		0.63	0.26	0.31		0.50	0.21

^a^VLW_DALY_: value of lost welfare–disability-adjusted life years.

^b^VLW_YLL_: value of lost welfare–years of life lost.

### Productivity Loss Based on YLLs

Corresponding estimates based on YLL are detailed in Table 3. Nationally, CHD-related productivity loss accounts for 1.35% of GDP using the US DOT benchmark, 0.91% with the OECD benchmark, and 0.72% under the national wage model. According to the national wage benchmark, East Nusa Tenggara records the highest VLW-to-GDP ratio at 3.64% (males=5.16% and females=3.03%), followed by West Sulawesi (2.92%), Maluku (2.84%), Gorontalo (2.28%), and West Nusa Tenggara (1.93%). In contrast, Jakarta (0.08%), East Kalimantan (0.16%), Riau (0.26%), and Riau Islands (0.27%) exhibit much lower VLW-to-GDP ratios. As populous provinces, West Java (0.90%), Central Java (0.67%), and East Java (0.40%) fall at or below the national average.

### Gender Disparity

**Figure 1:**
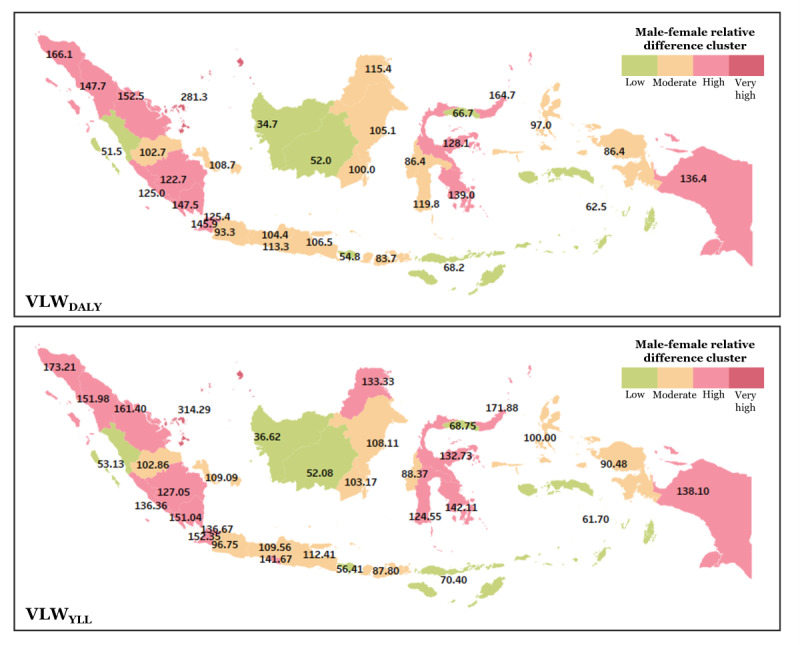
Male-female relative difference showing gender disparities across Indonesia, estimated from VLW_DALY_ (value of lost welfare–disability-adjusted life years) and VLW_YLL_ (value of lost welfare–years of life lost).

High gender disparities across Indonesian provinces, revealed by the male–female relative difference, are presented in Figure 1 and Multimedia Appendix 2. Based on the gender disparity in VLW_DALY_ (value of lost welfare–disability-adjusted life years), provinces such as Riau Islands (281.25%) stand out in the very high disparity category. A significant number of provinces fall into the high disparity cluster, including Aceh (166.10%), Banten (145.86%), Lampung (147.52%), North Sumatra (147.71%), and Papua (136.43%), reflecting consistently male-dominated VLW in both western and eastern regions. The moderate disparity group encompasses provinces such as Central Java (104.41%), East Java (106.51%), South Kalimantan (100%), and Yogyakarta (113.33%). Meanwhile, the low disparity cluster, with relative differences below 70%, includes Bali (54.76%), Central Kalimantan (52.00%), West Kalimantan (34.67%), Maluku (62.50%), and East Nusa Tenggara (68.22%).

The VLW_YLL_ (value of lost welfare–years of life lost) gender disparity reveals substantial variation in male-to-female differences across Indonesia. The most extreme case is seen in the Riau Islands, with a relative difference of 314.29%. A large group of provinces falls into the high disparity category, including Aceh (173.21%), Banten (152.35%), North Sumatra (151.98%), Lampung (151.04%), Papua (138.10%), and Yogyakarta (141.67%). The moderate disparity group features provinces such as Central Java (109.56%), East Java (112.41%), South Kalimantan (103.17%), and North Maluku (100%). In contrast, the very low disparity cluster includes Bali (56.41%), Central Kalimantan (52.08%), West Kalimantan (36.62%), and East Nusa Tenggara (70.40%). The gender disparity patterns in VLW_DALY_ and VLW_YLL_ for CHD are consistent. However, North Kalimantan and Yogyakarta are 2 provinces categorized as moderate disparity in the VLW_DALY_ but rise to high disparity in the VLW_YLL_.

### Geographical Disparity

**Figure 2:**
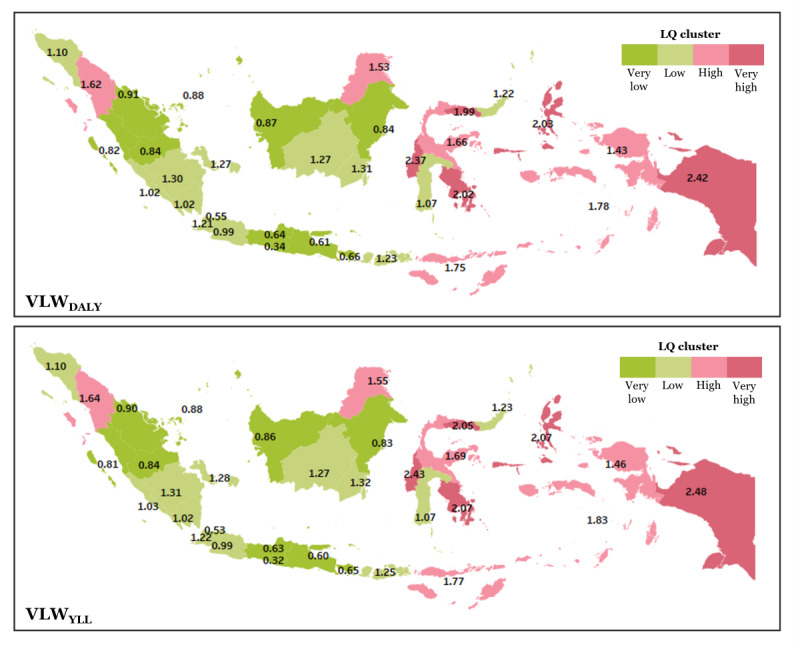
Location quotient (LQ) showing the economic burden of congenital heart disease (CHD) relative to the share population across Indonesia, estimated from VLW_DALY_ (value of lost welfare–disability-adjusted life years) and VLW_YLL_ (value of lost welfare–years of life lost). An LQ greater than 1 indicates a disproportionately higher economic burden relative to population share.

**Figure 3:**
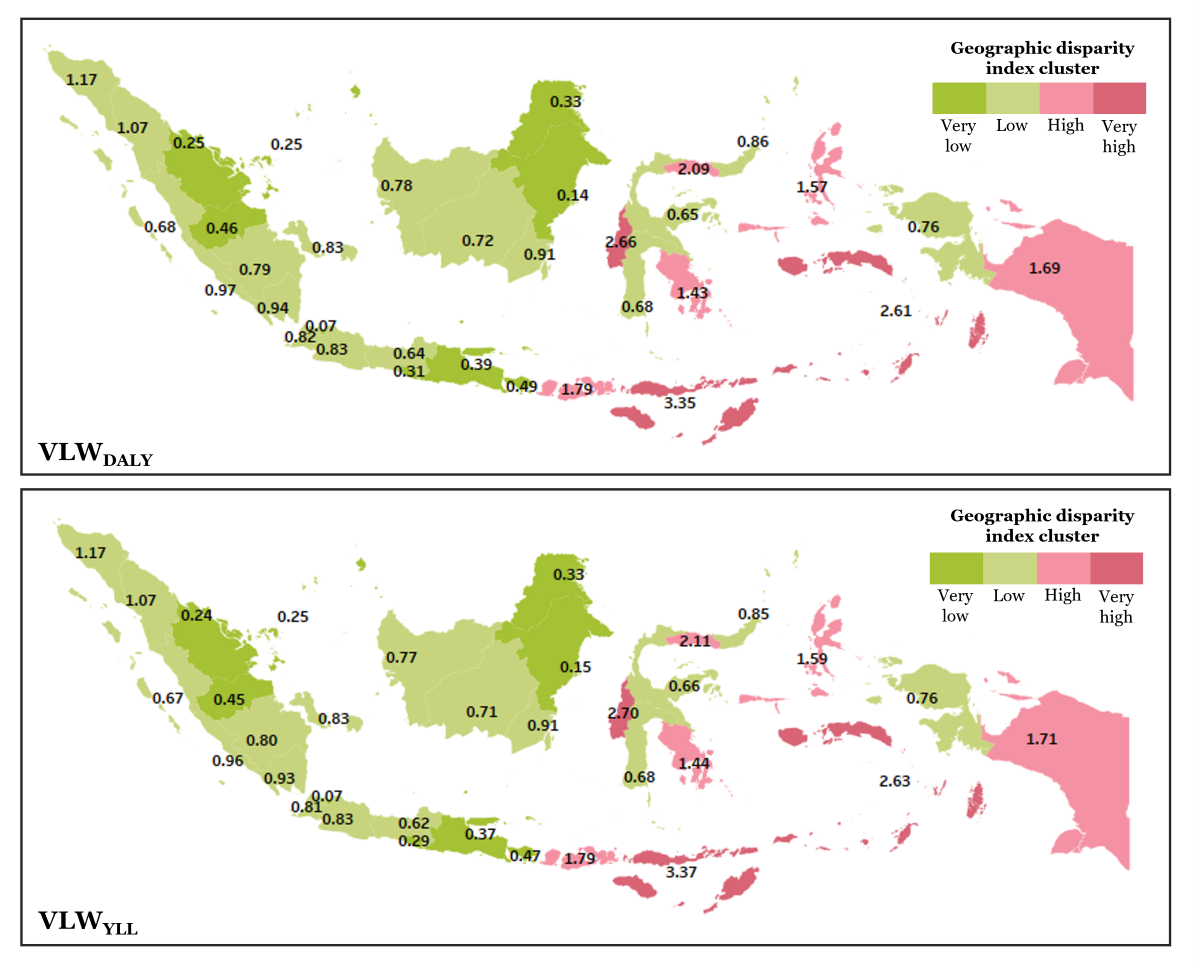
Geographic disparity index of the CHD economic burden across Indonesia, estimated from VLW_DALY_ (value of lost welfare–disability-adjusted life years) and VLW_YLL_ (value of lost welfare–years of life lost).

The geographical analysis of economic CHD burden in Indonesia reveals notable spatial disparities in the VLWs (Figures 2 and 3; Multimedia Appendix 2). Based on the VLW_DALY_ LQ, most provinces fall into the low (LQ=1.0-1.3) and very low (<1.0) categories, indicating that their share of CHD-related welfare loss is roughly proportional—or even lower—than their population size. Notably, Yogyakarta (0.34), Jakarta (0.55), East Java (0.61), Central Java (0.64), and Bali (0.66) fall under the very low group. In contrast, provinces such as Central Sulawesi (1.66), East Nusa Tenggara (1.75), Maluku (1.78), and North Sumatra (1.62) are in the high group, while Gorontalo (1.99), North Maluku (2.03), Papua (2.42), Southeast Sulawesi (2.02), and West Sulawesi (2.37) are in the very high group. Similarly, the VLW_YLL_ LQ follows a consistent trend. Provinces with very low LQs include Yogyakarta (0.32), Jakarta (0.53), East Java (0.60), Central Java (0.63), and Bali (0.65). Meanwhile, high disparity is observed in Central Sulawesi (1.69), East Nusa Tenggara (1.77), Maluku (1.83), and North Sumatra (1.64). The very high group again includes Gorontalo (2.05), North Maluku (2.07), Papua (2.48), Southeast Sulawesi (2.07), and West Sulawesi (2.43).

VLW_DALY_-to-GDP disparity revealed that the majority of provinces in Indonesia (20 of 34) fall into the “low disparity” group, with ratios ranging from 0.636 in Central Java to 1.174 in Aceh, indicating that while the burden exists, it is proportionate to local GDP. Provinces such as Banten (0.816), North Sumatra (1.067), and West Java (0.834) also belong to this group. The “very low disparity” cluster includes 9 provinces, such as Jakarta (0.072), East Kalimantan (0.143), Riau and Riau Islands (both 0.251), and Yogyakarta (0.314), showing minimal economic strain from CHD-related welfare loss. In contrast, 5 provinces fall into the “high disparity” category, including West Nusa Tenggara (1.793), Papua (1.694), and North Maluku (1.569), where the economic burden is disproportionately high relative to GDP. Most critically, East Nusa Tenggara (3.352), Maluku (2.608), and West Sulawesi (2.662) are classified under “very high disparity.”

Even though similar to the VLW_DALY_-to-GDP, where the indices of most provinces (21 of 34) fall into the “low disparity” category, discrepancies exist in the trend. The value was lowest in Central Java (0.620) and in highest in Aceh (1.166). Provinces such as Banten (0.814), Lampung (0.935), and North Sumatra (1.073) belong to this group. The “very low disparity” cluster includes 9 provinces, including Jakarta (0.074), East Kalimantan (0.148), Riau (0.241), and Yogyakarta (0.287). Conversely, 5 provinces are classified under “high disparity,” with indices ranging from 1.444 in Southeast Sulawesi to 2.110 in Gorontalo, indicating a disproportionate burden relative to their GDP. Notably, 3 eastern provinces—East Nusa Tenggara (3.369), Maluku (2.628), and West Sulawesi (2.702)—fall into the “very high disparity” group.

## Discussion

### Overview

This study estimated the economic burden of CHD across all 34 Indonesian provinces, highlighting substantial national losses as well as marked regional and gender disparities. The VLW-to-GDP ratio across provinces exceeds 1 million (×10^–^⁵), underscoring the significant burden of premature mortality and disability associated with CHD. This widespread productivity loss represents not only an urgent public health challenge but also a constraint on equitable economic development. High-income provinces such as Jakarta, East Kalimantan, and the Riau Islands reported some of the largest absolute VLW estimates, frequently exceeding US $10 billion even under conservative wage-based valuations. However, their VLW-to-GDP ratios remain modest due to their larger economic output. Nonetheless, the absolute magnitude of these losses highlights a substantial, and perhaps underappreciated, burden of disease. The persistence of CHD-related productivity losses in economically advantaged provinces points to systemic inefficiencies in health care delivery; despite higher income levels and relatively advanced health infrastructure, these regions continue to experience significant losses in human capital.

Overall, the CHD-related VLW-to-GDP ratio remains under 5% in most provinces, suggesting a modest economic burden nationally. However, the relative burden is considerably higher in economically vulnerable eastern provinces, where limited health infrastructure and geographic barriers amplify the impact of premature mortality and disability. In contrast, higher-income provinces such as West Java, East Java, North Sumatra, and Jakarta reported the highest absolute VLW_DALY_ estimates, ranging from US $0.79 billion to US $1.60 billion under wage-based valuations. However, due to their stronger economic output, these areas exhibited modest VLW-to-GDP ratios. These provinces also had LQ values <1.0, suggesting their share of VLW is proportionate to—or even smaller than—their population size. Nonetheless, the absolute magnitude of these losses—despite higher health infrastructure—suggests systemic inefficiencies in health care delivery and delays in diagnosis or treatment within CHD care pathways.

This phenomenon aligns with the Preston Curve, in which higher GDP does not automatically translate into better health outcomes without commensurate health system performance [26]. Similar patterns have been observed in coronary heart disease, where economic losses persisted despite macroeconomic gains, estimated at US $33.3 billion nationally [27]. In this context, the high VLW in affluent provinces may reflect missed opportunities in prevention, early detection, or effective intervention—rather than resilience. Conversely, resource-limited provinces, particularly in eastern Indonesia—East Nusa Tenggara (3.35%), Maluku (2.61%), and West Sulawesi (2.66%)—though reporting lower absolute VLW (US $0.1-US $0.3 billion), experienced disproportionately high relative burden and LQ values (2.4-2.7). This suggests structural fragility: limited pediatric cardiology services, fragmented referral networks, and geographic barriers all magnify the consequences of delayed care [28,29]. The findings thus illuminate a dual burden: in wealthier, urbanized provinces, absolute VLW losses suggest inefficiencies in service delivery, whereas in poorer, rural areas, high VLW-to-GDP ratios and elevated LQ values highlight acute economic vulnerability from preventable losses. Collectively, these patterns underscore the need for nuanced, region-specific policy responses.

### Burden Patterns: DALYs Versus YLLs

Our provincial analysis in this study revealed that the difference between VLW_DALY_ and VLW_YLL_ is small—generally under 5% across most regions, with only Yogyakarta exceeding a 10% gap. This finding indicates that premature mortality remains the dominant contributor to productivity loss from CHD in Indonesia, rather than chronic morbidity. It also suggests that while rehabilitation and long-term care are important, the most immediate policy priority in many provinces remains the prevention of early deaths through improved detection and timely intervention. Many infants do not survive CHD—largely due to late diagnosis, limited surgical access, and insufficient intensive neonatal and postoperative care [4,30]. In remote regions, care-seeking is often delayed due to low health literacy, poor transportation, financial hardship, and cultural beliefs [28,31]. As a result, many congenital anomalies remain undetected until they cause serious complications or early death [31]. In line with the previously reported economic evaluations of congenital anomalies, strengthening early detection and intervention is thus essential to reduce VLW_DALY_ [27]. In Bali, Central Java, East Java, or Jakarta, persistent premature mortality likely affects marginalized subgroups within otherwise well-equipped provinces. Similar patterns have been reported in the United States, where CHD mortality remains unequally distributed despite overall health system strength [32,33]. Yogyakarta’s relatively higher DALY-to-YLL difference likely reflects both effective early detection and follow-up care systems [34,35], as well as a sociodemographic structure with a lower concentration of underserved populations compared to megacities such as Jakarta [36].

### Geographic Divide

This study introduces 2 complementary indicators of geographic burden: the VLW-to-GDP disparity index and the VLW LQ. While both indicators reveal a consistent east–west divide, they offer distinct perspectives. The VLW-to-GDP disparity index classifies 20 of 34 provinces as “low disparity” and 9 as “very low,” indicating that in most regions, the CHD-related economic burden remains relatively proportional to local GDP. However, East Nusa Tenggara (3.35%), Maluku (2.61%), and West Sulawesi (2.66%) fall into the “very high disparity” cluster, pointing to severe strain relative to their limited economic capacity. These findings support previous reports highlighting chronic health care underdevelopment and financial vulnerability in these eastern provinces [37,38]. Residents of Maluku are significantly less likely than even those in Papua to use hospital services, with an adjusted odds ratio (OR) of 0.827 (95% CI 0.820-0.835), reflecting both structural and access-related barriers to care [38]. Such disparities in hospital use emphasize how systemic underutilization of formal health services may contribute to both the progression of undiagnosed CHD and increased long-term economic burden in these regions. In addition, comparable regional gradients have been observed in China’s poverty and population distribution using remote sensing–based spatial models [12,13]. These parallels highlight how geospatial approaches can uncover hidden structural inequities, reinforcing the need for province-specific strategies in Indonesia.

The LQ, by contrast, measures each province’s share of the national CHD-related burden relative to its population size. Provinces such as Gorontalo, North Maluku, Papua, Southeast Sulawesi, and West Sulawesi had LQ values exceeding 2.0, suggesting that their per capita contribution to national VLW is disproportionately high. Other regions, including East Nusa Tenggara, Maluku, North Kalimantan, and North Sumatra, also fall into the “high” category. In contrast, economically stronger and densely populated provinces such as Jakarta, Yogyakarta, Central Java, and East Java show consistently low LQ values (0.32-0.64), indicating a lower per capita burden. This may reflect underlying disparities within otherwise well-resourced provinces, where marginalized or socioeconomically disadvantaged subpopulations continue to face barriers to timely diagnosis and treatment. Previous research in high-income contexts, including the United States, has similarly demonstrated that CHD-related mortality is unequally distributed across populations, with persistent service gaps and socioeconomic deprivation contributing to avoidable deaths [32,33].

When DALY-based and YLL-based disparity indices are compared, the results show broadly similar geographic patterns but also important differences. Both measures consistently flag East Nusa Tenggara, Maluku, and West Sulawesi as high-disparity regions, while Jakarta, East Kalimantan, Riau, and Yogyakarta appear in the very low cluster across both metrics. However, DALYs, which include YLDs, tend to be higher in provinces such as West Nusa Tenggara, where survival is often accompanied by long-term morbidity. Conversely, Gorontalo and North Maluku exhibit higher YLL-based indices, suggesting early death as the dominant burden component.

### Gender Disparities and Economic Impact Severity

Across all provinces and valuation models, males consistently contributed a greater share to the total VLW than females, underscoring persistent gender-based disparities in the economic impact of CHD. This pattern aligns with prevailing socioeconomic structures in Indonesia, where men dominate formal labor markets and are typically regarded as primary income earners [39]. As a result, premature death or long-term disability in males translates into greater economic loss, particularly in contexts where male productivity is central to household income and community stability [40].

In this study, the magnitude of this disparity varies considerably by province. Riau Islands exhibited the most extreme male-dominant burden, with a VLW-to-GDP gender difference of 281.25% (DALY-based) and 314.29% (YLL-based). Several other provinces, such as Aceh, Banten, North Sumatra, Lampung, and Papua, consistently fall into the high-disparity category (≥130% higher in males), spanning both western and eastern regions. In contrast, provinces such as Bali, Central Kalimantan, West Kalimantan, Maluku, and East Nusa Tenggara showed much lower disparities, with male VLW contributions below 70% higher than female, suggesting either more balanced socioeconomic structures or more equitable access to care.

While the overall pattern is consistent across DALY- and YLL-based measures, some provinces, such as Yogyakarta and North Kalimantan, demonstrated higher disparity in YLL-derived estimates, indicating that male premature mortality plays a more dominant role than long-term male disability therein. Given that CHD prevalence also remains higher in males (0.96%) than in females (0.72%) [2], targeted policy responses must consider sex-specific vulnerabilities to reduce both health and economic consequences, particularly in provinces with extreme gender disparity.

### Implications and Policy Recommendations

Our dual analysis using VLW-to-GDP disparity indices and LQ reveals distinct yet overlapping geographical patterns, underscoring the need for a province-specific strategy that balances financial, systemic, and service-based interventions. In lower-resource provinces, elevated VLW_DALY_ reflects the economic consequences of survival without adequate recovery, highlighting fragmented care pathways and limited rehabilitative infrastructure. Conversely, in more affluent regions, high VLW_YLL_ underscores that income alone does not ensure early diagnosis or optimal outcomes. These findings point to persistent system inefficiencies that allow both preventable mortality and long-term disability to persist across disparate socioeconomic contexts.

Timely diagnosis is central to effective CHD management, yet screening remains inconsistently implemented nationwide. Although feasibility studies in Yogyakarta have demonstrated successful integration of pulse oximetry into newborn care [4], national scale-up has been hindered by logistical and workforce barriers. School-based screening using auscultation and electrocardiography has also shown promise [3] but remains fragmented and pilot-driven. A recent national review highlights the absence of a cohesive CHD screening strategy, contributing to diagnostic delays, often until complications such as pulmonary hypertension or irreversible cyanosis arise [41]. Screening strategies should be prioritized in light of existing evidence showing that universal neonatal pulse oximetry screening is not only effective but also highly cost-effective [42]. Beyond clinical access, the economic burden of CHD is not gender-neutral. As findings of this study suggest, male children represent a larger share of projected productivity losses due to their expected roles in the formal labor market. Provinces such as Riau Islands, Aceh, and Papua reveal extreme disparities in male-to-female VLW, suggesting that male early mortality or disability carries greater economic implications, especially in socioeconomically vulnerable areas.

To effectively reduce geographic inequities, interventions must be tailored to the burden profile of each province. Regions such as East Nusa Tenggara, Maluku, and West Sulawesi, which show both high local strain (limited resources) and moderate contribution to the national CHD-related economic burden, warrant comprehensive support. These provinces present a strategic opportunity for high-return investment: their low baseline health care capacity means that even modest improvements in screening, referral, or access to surgical services can yield substantial health gains [43,44]. As previously reported, deep learning–assisted prenatal ultrasound is highly cost-effective, generating more than 1200 additional quality-adjusted life years at a cost of just US $1720 per quality-adjusted life year [45]. Moreover, alleviating the burden in these areas reduces both localized economic strain and the national VLW [46]. Targeted interventions here not only address vulnerability but also improve overall system efficiency, making them particularly cost-effective. In contrast, provinces such as West Nusa Tenggara and Bengkulu, despite contributing less to the national burden, still experience significant local impact. These areas require strengthened local infrastructure and the continuation of full coverage by Indonesia’s Universal Health Coverage system, covering surgery, diagnostics, and long-term follow-up, to shield households from out-of-pocket costs and prevent untreated cases from progressing into costly long-term disabilities [47].

Other areas, such as Papua, North Maluku, and Gorontalo, contribute significantly to the national VLW through high premature mortality, suggesting the need for enhanced early detection, community engagement, and strengthened referral systems. However, such efforts will remain insufficient without addressing the chronic shortage and unequal distribution of pediatric cardiologists and cardiac surgeons in these regions [48]. Evidence from cost-effectiveness studies consistently supports the value of expanding surgical access in LMICs [49]. Strengthening pediatric surgical capacity in Indonesia would therefore not only reduce preventable mortality but also represent an efficient use of limited health resources.

To reduce preventable mortality and economic loss, targeted investments are needed to expand local specialist capacity, develop regional cardiac centers, and implement remote consultation systems such as telecardiology [50,51]. Task-shifting approaches, such as training nurses or midwives to perform point-of-care cardiac ultrasound, have demonstrated effectiveness in resource-limited settings by enabling earlier detection and timely referral for CHD [52]. While challenges remain, including training costs, limited internet connectivity, and unstable electricity supply, these models have been proven to address the limited specialist availability [52].

Meanwhile, well-performing provinces such as Jakarta, Central Java, and Yogyakarta can serve as national innovation hubs to accelerate progress in CHD care. These regions are well-positioned to support interprovincial partnerships and lead pilot projects for digital health integration, including the development of artificial intelligence (AI)–based models for early CHD detection and risk stratification [53-55]. Their advanced clinical infrastructure allows for the implementation of high-impact interventions, such as zero-fluoroscopy atrial septal defect closures [56], transesophageal echocardiography–guided balloon-assisted percutaneous closures in complex cases (including during pregnancy) [57], and percutaneous palliative stenting for adults with unrepaired Tetralogy of Fallot [58]. Furthermore, these provinces can spearhead trials on advanced therapies, remote robotic-assisted surgery, and AI-assisted procedural planning [59,60], while also serving as training centers for pediatric cardiologists and interventional specialists [48].

### Limitations

This study is subject to several limitations, chiefly stemming from its reliance on the GBD 2019 dataset. While the GBD framework provides standardized, internationally comparable estimates of disease burden, its modeled nature may not adequately reflect the subnational heterogeneity in CHD prevalence, health care access, and mortality reporting that characterizes Indonesia’s archipelagic geography. Provinces with limited health surveillance infrastructure, particularly in eastern and remote regions, may exhibit substantial discrepancies between modeled and actual disease burden, introducing uncertainty into provincial-level estimates. Furthermore, the use of age-standardized data, though essential for international comparability, may obscure local demographic nuances, such as regional fertility rates, health-seeking behaviors, and differential survival trajectories—factors particularly salient in the context of pediatric conditions.

In addition, the valuation methods used, although grounded in established economic frameworks (eg, US DOT, OECD, and wage-based models), may inadequately account for informal labor contributions, gendered economic roles, and intrahousehold productivity, all of which are significant in the Indonesian context. Nevertheless, the GBD remains among the most comprehensive sources for cross-provincial disease burden estimation and provides a rigorous foundation for quantifying the economic impact of CHD across diverse settings. The triangulation of estimates across multiple valuation models further strengthens the robustness of the findings and highlights important spatial and sociodemographic disparities.

### Conclusion

Our findings reveal that CHD imposes a macroeconomically nonsignificant burden in Indonesia, accounting for up to 1.35% of GDP using the highest valuation model. Similar to patterns observed in other LMICs, this economic impact is primarily driven by premature death. Across provinces, the gap between DALY- and YLL-based estimates is typically under 5%, indicating that YLLs dominate the productivity losses from CHD. Notably, gender disparities are prominent, with provinces such as the Riau Islands showing very high male–female relative differences in both DALY- and YLL-based losses. In Yogyakarta and North Kalimantan, the disparity is more pronounced in YLL-derived estimates, suggesting that premature mortality among males is a key contributor. Geographic disparities further reveal that provinces such as East Nusa Tenggara, Maluku, and West Sulawesi—which experience both high local strain and moderate national burden—warrant comprehensive and fundamental support. Meanwhile, provinces with low VLW-to-GDP ratios, including Jakarta, Central Java, and Yogyakarta, can serve as innovation hubs, facilitating specialist training, piloting AI-assisted interventions, and supporting interprovincial knowledge exchange to reduce national inequities.

## Appendix

Multimedia Appendix 1 Regional DALY and YLL Burden, Population Size, and GDP per Capita.

Multimedia Appendix 2 Value of Lost Welfare (VLW), Gender Disparity, and Ratio Estimates Based on DALY and YLL Metrics.

Multimedia Appendix 3 Graphical abstract.

## Abbreviations

AI: artificial intelligence
BPS: Badan Pusat Statistik
CHA: congenital heart anomaly
CHD: congenital heart disease
DALY: disability-adjusted life year
GBD: Global Burden of Disease
GDP: gross domestic product
LMIC: low- and middle-income country
LQ: location quotient
OECD: Organisation for Economic Co-operation and Development
OR: odds ratio
US DOT: US Department of Transportation
VLW: value of lost welfare
VLWDALY: value of lost welfare–disability-adjusted life years
VLWYLL: value of lost welfare–years of life lost
VSL: value of a statistical life
VSLY: value of a statistical life year
YLD: year lived with disability
YLL: year of life lost

## Data Availability

All data generated or analyzed during this study are included in this study and its supplementary information files.
