# Supplementary figures and images for: Monetary Valuation of Congenital Heart Disease in Indonesia: Economic Modeling Study
Source: JMIR Pediatr Parent. 2025 Nov 19;8:e80696. doi: 10.2196/80696 (PMC12676220; doi:10.2196/80696)

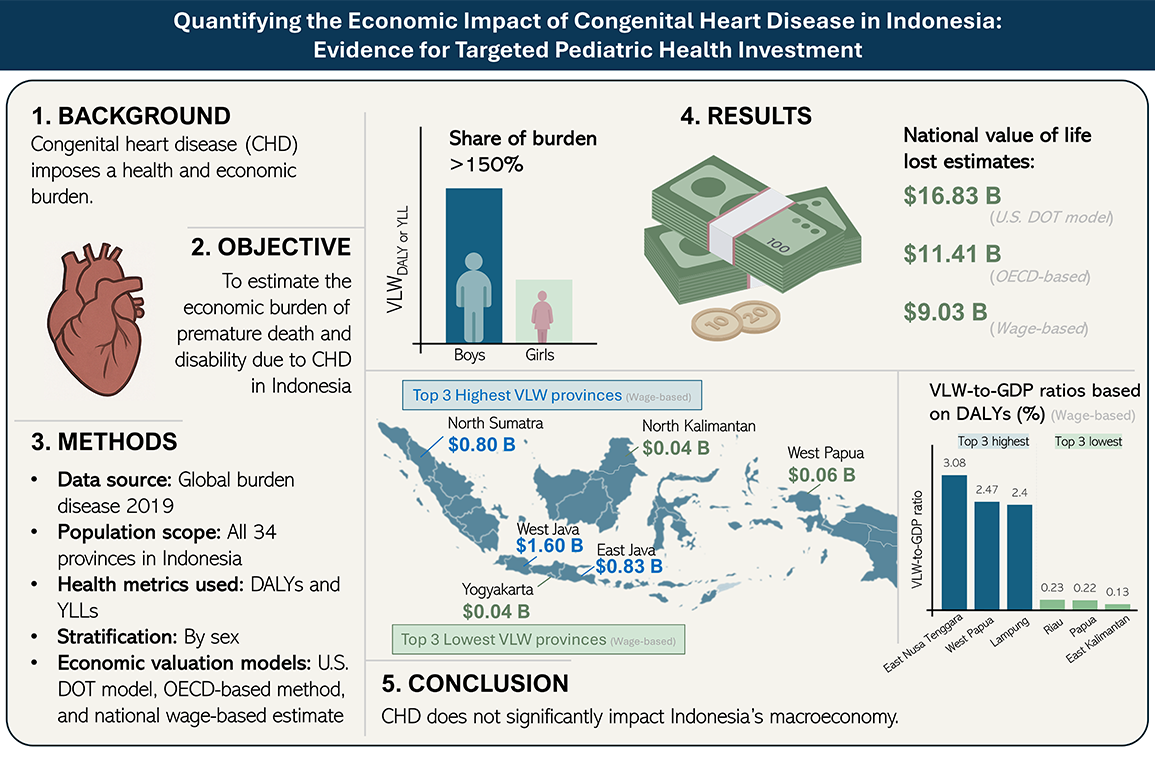

Supplement: Multimedia Appendix 3 [file pediatrics_v8i1e80696_app3.png]
